# An Augmented Reality Serious Game for Learning Intelligent Wheelchair Control: Comparing Configuration and Tracking Methods

**DOI:** 10.3390/s22207788

**Published:** 2022-10-13

**Authors:** Rafael Maio, Bernardo Marques, João Alves, Beatriz Sousa Santos, Paulo Dias, Nuno Lau

**Affiliations:** 1 IEETA, DETI, Campus Universitário de Santiago, University of Aveiro, 3810-193 Aveiro, Portugal; 2 DigiMedia, DeCA, Campus Universitário de Santiago, University of Aveiro, 3810-193 Aveiro, Portugal

**Keywords:** remote collaboration, augmented reality, Industry 4.0, maintenance authoring tool, visual characteristics, human-centered design, user study

## Abstract

This work proposes an augmented reality serious game (ARSG) for supporting individuals with motor disabilities while controlling robotic wheelchairs. A racing track was used as the game narrative; this included restriction areas, static and dynamic virtual objects, as well as obstacles and signs. To experience the game, a prior configuration of the environment, made through a smartphone or a computer, was required. Furthermore, a visualization tool was developed to exhibit user performance while using the ARSG. Two user studies were conducted with 10 and 20 participants, respectively, to compare (1) how different devices enable configuring the ARSG, and (2) different tracking capabilities, i.e., methods used to place virtual content on the real-world environment while the user interacts with the game and controls the wheelchair in the physical space: C1—motion tracking using cloud anchors; C2—offline motion tracking. Results suggest that configuring the environment with the computer is more efficient and accurate, in contrast to the smartphone, which is characterized as more engaging. In addition, condition C1 stood out as more accurate and robust, while condition C2 appeared to be easier to use.

## 1. Introduction

The human–robot interaction (HRI) field is vast; there are many different robots used in the most diverse areas in which robotics are applied. However, integrating these robots into human society on a daily basis (to improve human productivity) is not linear [1,2,3]. The same occurs with the robotic wheelchair branch of robotics. Robotic wheelchair usage is growing rapidly (forecasted to expand 8% by 2028 (grandviewresearch.com/industry-analysis/wheelchair-market (accessed on 12 September 2022))), thanks to the longer life expectancy and the new features these can bring to individuals with motor disabilities [4,5,6,7,8,9]. However, due to a lack of experience, some people do not find the interaction straightforward, and have difficulties acclimating to its manipulation (and, therefore, require initial training [5,7,8,9]).

Augmented reality (AR) technologies can be used in such scenarios, complementing real environments with additional layers of virtual information [10,11,12,13,14]. AR is commonly used to assist HRI, increasing engagement and providing richer experiences [15,16,17]. Prior studies had success employing AR to support HRI, integrating digital information in the robot workspace, simplifying the interaction, and removing the need for understanding the vast theory behind the robots [18,19,20]. Moreover, pervasive AR extends this concept through experiences that are continuous in space, being aware of and responsive to the user’s context and pose [21,22,23], changing the way users interact with their surroundings [24].

Several studies investigated the control of motorized wheelchairs, the vast majority being on multimodal interfaces for their control: providing guidance with eye-tracking systems [25], electrooculogram (EOG) signals [26,27], head movements [28], applying voice-control commands [29], adopting autonomous traveling, and applying machine learning on the data obtained by the sensors integrated into the wheelchair [30,31]. These studies are mostly solutions for individuals with hand impairments, who do not possess the required capabilities to use the motorized wheelchair-integrated joystick, and needing additional technology to assist their maneuvering. By contrast, few studies have proposed methods to support the learning and training of regular robotic wheelchairs. Traditional approaches use real-world scenarios, despite being simpler, and contain real obstacles and doorways (https://rehabpub.com/conditions/neurological/stroke-neurological/optimizing-power-wheelchair-use-mobility-training/, 12 September 2022); thus, collisions can occur, damaging materials, the motorized wheelchair, or even injuring the user [4]. Other methods were designed to support the learning of robotic wheelchair maneuvers, using simulated environments in virtual reality (VR) [32,33] and adopting AR environments, enabling virtual assistance within the real world [7,8]. Zolotas and Demiris [8] (in continuation of a previous study [7]) presented an AR application for the HMD HoloLens (https://learn.microsoft.com/en-us/hololens/hololens1-hardware, 12 September 2022) to resolve the misalignment between their actions and the respective internal models of the robot. For this purpose, they included three visual clues in the system: spheres to highlight collisions in the physical environment (increasing the contextual awareness of users); a mini-map panel forecasting the robot’s estimated poses after applying input commands; and an additional display to supplement the rear view with a virtual wheelchair avatar during backward movements. Results and conclusions from their studies indicate the benefits of learning the robotic wheelchair control using AR, as users were faster, more confident, and understood what they were doing wrong.

While the primary goal of standard games is entertainment, the serious game‘s main purpose is to use fun and/or competition to add pedagogical value to the game. Thus, people can enjoy the time spent learning or training concrete activities through serious storytelling. Activities supported by serious games include: education, healthcare, defense, city planning, emergency management, engineering, and others [34,35,36,37]. There are benefits in AR engagement in games, i.e., exciting gaming experiences as it encourages people to move more and wander outside, breathing fresh air in an AR play space, whereas non-AR experiences limit these users to a screen. It could also lead to social and emotional benefits [38,39,40]. On the other hand, studies that have integrated AR into serious games demonstrated the pedagogic enhancement that this merge produces [41,42,43,44]. As the topic of this study is health and exercise therapy for people with motor disabilities, AR is an interesting technology that could support serious games in this field due to the physical movements that it entails.

This paper proposes an ARSG with two tracking modalities: motion tracking using cloud anchors and offline motion tracking, focused on assisting the learning process of controlling a robotic wheelchair. A configuration tool to set up the AR environment is presented. Additionally, a visualization tool was developed to discover maneuvering mistakes and how to improve the robotic wheelchair control, visualizing the user actions performed during the game. The configuration tool was evaluated through a user study with 10 participants. We evaluated which device (smartphone or computer) was preferable to perform the environment configuration. Both versions of the ARSG were also evaluated through a user study with 20 participants, focusing on usability and navigation of the robotic wheelchair in a physical environment enhanced with virtual content.

The remainder of the document is organized as follows: First, the ARSG is described, including its concept and architecture, as well as the technical implementation and communication between the implemented modules. Then, two user studies are presented, focusing on: 1—comparing configuration methods using different devices; 2—comparing tracking methods. Then, their results are presented and discussed. Finally, the concluding remarks and ideas for future work are presented.

## 2. An Augmented Reality Serious Game for Robotic Wheelchair Control

**Figure 1:**
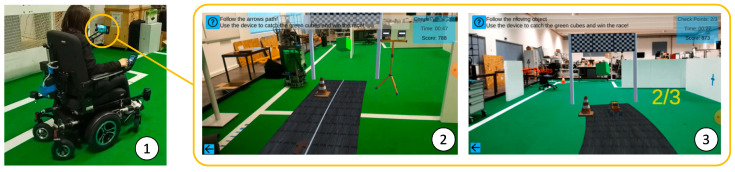
User experiencingthe augmented reality serious game (ARSG): 1—set up used for controlling the robotic wheelchair; 2—following the white line in the middle of the road; 3—following the path of the moving object (car) until it stops.

The proposed game helps users learn how to control robotic wheelchairs. It takes advantage of the capacity of AR to overlap additional layers of virtual content in a real-world environment, as well as the serious game’s learning capabilities to capture the user’s attention/interest while moving around the environment. The requirements used for the development of the ARSG were defined with support from rehabilitation professionals in the scope of a research project (https://www.intellwheels.com/en/consortium/team/, 12 September 2022). The ARSG concept was built to answer the needs of individuals with motor disabilities to master robotic wheelchair control. The narrative follows a racing game, composed of a curved track with one to various passage points that need to be passed by during the race while controlling the robotic wheelchair with its integrated joystick. To enrich the game, additional constraints were considered: deflecting from virtual obstacles, obligation to maintain the wheelchair inside the road area, a stop sign and a spotlight to look away, training user reactions, and decision-making. The required actions illustrated in the game narrative reflect real-world activities that can better prepare users for dealing with daily scenarios. There are two modes to play the ARSG: 1—following a static line in the middle of the road or 2—pursuing a moving virtual object (car) (see Figure 1(1–2), respectively).

### 2.1. Technologies

Requiring the navigation over an indoor scenario, the most suitable software development kit (SDK) for our use case was ARCore (developers.google.com/ar (accessed on 12 September 2022)), which is reported to have great feedback on its motion tracking and environmental understanding features [45,46]. This SDK runs on handheld devices, facilitating the first contact with the application and representing a cheaper option for the final user (than, for example, head-mounted displays (HMDs)). ARCore integrates the following relevant features for this study: motion tracking to estimate the device pose, using the simultaneous localization and mapping (SLAM) method, which detects visually distinct features to compute its change in location while moving, combined with the device’s inertial measurement unit (IMU); environmental understanding is responsible for detecting planes, using agglomerate feature points strongly connected in common surfaces and storing these planes to build the world model; depth, allowing the occlusion of real and virtual objects; anchors fix the locations and orientations in the real world, ensuring that virtual objects connected to anchors remain stable over time; cloud anchors are variants of standard anchors; however, they offer persistence and collaboration. Previous studies have demonstrated that ARCore SDK is an adequate tool for applications requiring indoor navigation [47,48,49].

### 2.2. Architecture

**Figure 2:**
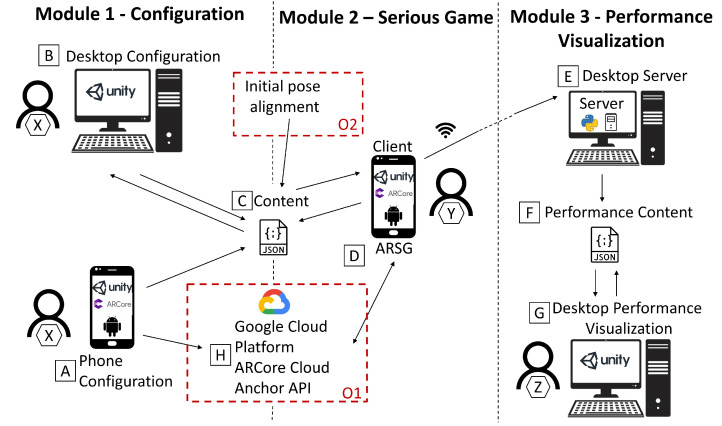
Architecture overview: A—configuration of the ARSG using the smartphone device; B—configuration of the ARSG using the computer; C—file generated in the configuration process containing the virtual information; D—visualization and interaction with the ARSG; E—server receiving messages from the ARSG; F—file with the user actions while playing the ARSG; G—display of user actions; H—API for storing cloud anchors; X—user performing the configuration; Y—user playing the ARSG; Z—user visualizing the user Y game performance; O1—requirement for motion tracking using the cloud anchor operating mode; O2—requirement for offline motion tracking operating mode.

Based on the features of ARCore and the benefits they can bring, two operating modes were implemented (Figure 2): O1—motion tracking using cloud anchors; O2—offline motion tracking.

**Figure 3:**
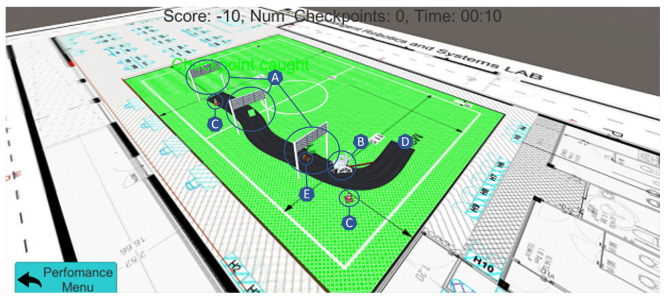
Visualization tool for revisiting the participant’s experiences and performances. The tool receives multiple variables from the smartphone attached to the robotic wheelchair, which enables the creation of a virtual representation/simulation of the ARSG. Overview: A—passage points; B—intelligible wheelchair model representing the robotic wheelchair location; C—obstacles; D—starting position; E—moving car.

Each operating mode is divided into three modules: configuration, serious game, and performance visualization, targeting distinct users (user X, Y, and Z). The **configuration module** is responsible for building the ARSG scenario (placing and storing the virtual objects around the real environment). This may be performed using a smartphone or a computer, implying a calibration process. The **serious game module**, typically used by a different user (user Y in Figure 2) from the one that uses the configuration component (user X in Figure 2), will hold and access the configured content, so it can position the virtual objects (and the cloud anchors, if operating mode O1 is used) in the real environment, assembling the AR scenario built during the configuration process. Finally, the **visualization module** logs the various user actions while playing. A message exchange system was elaborated adopting a client–server model through transmission control protocol/internet protocol (TCP/IP), where the client is the smartphone running the ARSG, responsible for sending messages via Wi-Fi, to a computer application, acting as a server. After being stored, these messages can be treated to display the user actions in a simulated environment (Figure 3).

One major difference between the two operating modes is the fact that O2 requires the alignment of the smartphone pose when starting the ARSG, to make it match the configuration starting pose. Plus, it does not need to store cloud anchors.

### 2.3. Module 1—Configuration

The configuration of the ARSG is mandatory, although it only needs to occur once for a specific real scenario (assuming that the scenario remains relatively stable and without significant changes over time). Its objective is to enable the placement of virtual objects in the real world in their intended positions to generate a complete AR experience. Furthermore, it requires persistence, so that a single configuration can be repeatedly used by the serious game module, and be easily adapted to other use cases, which demand the spreading of virtual objects in the real world.

**Figure 4:**
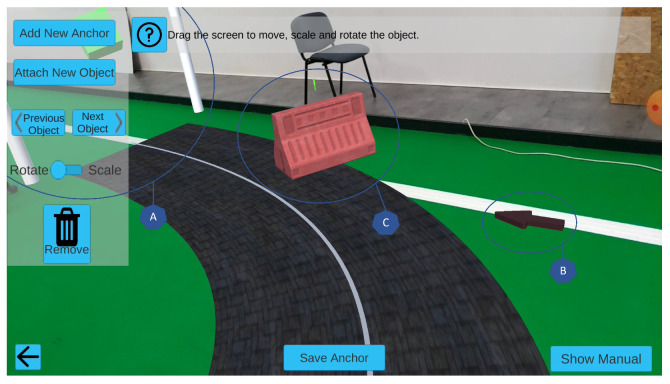
Configuration mode on a smartphone, illustrating how virtual objects can be positioned in the real-world environment. Overview: A—passage points; B—directional arrow; C—obstacle.

**Figure 5:**
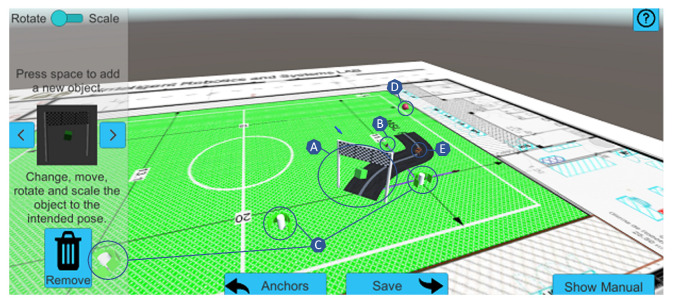
Configuration mode on a computer, illustrating how virtual objects can be positioned in a real-world environment. Overview: A—passage points; B—directional arrow; C—objects representing the cloud anchor locations; D—object representing the starting position; E—moving car.

For each operating mode, O1 and O2, we developed two methods of performing the configuration, using the smartphone (Figure 4) and/or the computer (Figure 5). In the operating mode O1, motion tracking using cloud anchors, the smartphone is used to place an anchor and map its surroundings to verify if the location has enough texture to store it persistently as a cloud anchor. Afterward, it is possible to associate virtual objects to the cloud anchor, through a set of modules previously defined, and apply them to the desired transformation (translation, rotation, and scaling) to position them as wished, using the drag, turn to rotate, and pinch/spread gestures.

Using the smartphone with operating mode O2 (offline motion tracking) the process is identical, but does not require storing cloud anchors; nevertheless, the configuration application has to be launched in a specific and well-known real-world pose. The approach using the computer together with operating mode O1 is based on the following steps: first, the smartphone is used to store the cloud anchors in the physical world, generating a file with each anchor’s relevant information and corresponding pose (relating to the initial smartphone position); then, this file is transferred to the computer application, where an architectural plan is used to represent the real scenario; it indicates where the application starts, objects representing the anchors placed in the real world are set in the architectural plan, and small adjustments are done, if necessary. The intended virtual objects can be associated with the closest anchor and the required transformations are performed using the mouse and the keyboard; with the virtual scenario built, a new file is created, containing the anchor information combined with the associated virtual object information.

On the other hand, in operating mode O2, the initial anchor placement using the smartphone is not required; however, in O1, it is not essential to indicate the smartphone’s initial position in the computer application (as the objects representing anchors can be manually positioned); in O2 it is required to indicate where the smartphone application will be launched.

Specific to the ARSG, we added the possibility to assemble a road using the configuration tool. The track construction was based on a Bézier curve, where the passage points represent the endpoints. In between each set of two endpoints, control points could be added to compose the curves.

### 2.4. Module 2—Serious Game

The second module is the virtual representation of the ARSG to be experienced in robotic wheelchairs. The operating mode used to mount the ARSG scenario depends on the operating mode used to configure it. For operating mode O1, the application begins by searching for the cloud anchors stored during the configuration. When the device’s camera points to the location where these were saved, the cloud anchors are recognized and every virtual object associated with the corresponding cloud anchor is instantiated and geometrically transformed to match its pose when configured. As for operating mode O2, it is necessary to launch the ARSG with the device situated in the same position and orientation as the one used during the configuration. Then, the virtual objects start to appear when they enter the smartphone’s field of view (FOV).

The game objective depends on the mode selected. When playing the *’follow the white line’* mode, users must follow the line in the middle of the road to maintain the wheelchair centered, as fast as possible. Regarding the *’pursue the moving car’* mode, the goal is to keep the wheelchair as close as possible to the car (moving at a constant speed). For both modes, users also have to comply with the remaining game features, to achieve the best scores possible.

## 3. User Studies

Two user studies were conducted. One to compare how devices influence the ARSG configuration and another to compare different tracking capabilities, i.e., methods used to place virtual content in the real-world environment, as these are fundamental aspects of the user experience.

### 3.1. Experimental Setup

**Figure 6:**
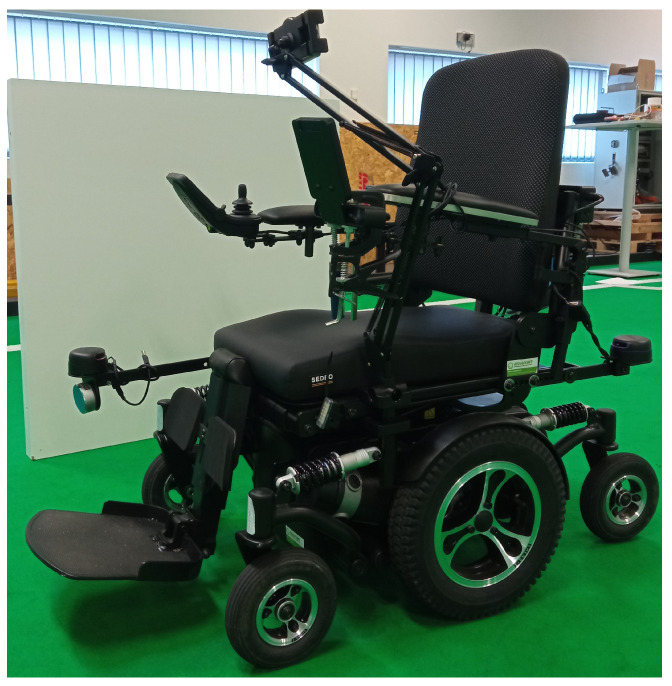
TA IQ MWD wheelchair with the smartphone attached.

Both studies were accomplished in a middle size league robotics soccer field. For the ARSG user study, the TA IQ MWD (ta-service.dk/uk/power-wheelchair/produkter/896-ta-iq-mwd (accessed on 12 September 2022)) motorized wheelchair (Figure 6) was used, including a controller, composed of a display, various buttons, and a joystick, enabling the control of the wheelchair’s speed and direction, it can also be used to obtain information concerning, for instance, the battery autonomy and speed. It allows five speed levels, with a maximum speed of 12.5 km/h, reachable in 3 s. Plus, the wheelchair has a turning radius of 45 cm to easily spin in tight spaces; all six wheels (four castor wheels and two driving wheels) are enabled with suspensions for smooth and comfortable trips.

A folding smartphone arm holder was fixed to the wheelchair, close to the left driving wheel, so that the device would rest in a stable position in front of the user, being able to adjust the smartphone position, depending on the user’s height and preference. Thus, the user does not need to hold the smartphone, which is imperative to wheelchair users also preventing them from obstructing the camera with their hands or fingers, as the ARCore performance significantly decreases when this occurs.

### 3.2. User Study 1—Comparing Devices for Configuration

The main goal of the first study was to compare in which device, smartphone, or computer, the user had more effectiveness and efficiency while performing the configuration (youtube.com/watch?v=2L5KqgJqVzU&ab_channel=RafaMaio (accessed on 12 September 2022)), as well as understand their preferences and possible difficulties. The task consisted of sequentially positioning four virtual objects over four semi-transparent virtual objects resting in predetermined positions, rotations, and scales. The first object required translation; the second object required translation, rotation, and scaling; the third and fourth objects required translation, rotation, scaling, and model switching. Two tasks were created: 1—configuration using the smartphone (Samsung Galaxy S7); 2—configuration using the computer (HP Pavilion 15-cs2016np). A within-subjects experimental design was used; the order of the tasks alternated among users to counterbalance the learning effects and participant performances. Opinions were registered. The following measures were considered: the time to place each object for efficiency, as well as the distance, orientation, and scaling difference between each configurable virtual object and the corresponding semi-transparent one for effectiveness. Participants were instructed on the experimental setup and tasks before providing informed consent. Then, they were introduced to the configuration tool and were given a time for adaptation until they felt comfortable, i.e., a training period to freely interact with the functions. Then, the tasks were performed; upon completion, participants answered a post-task questionnaire.

#### Results and Discussion

**Figure 7:**
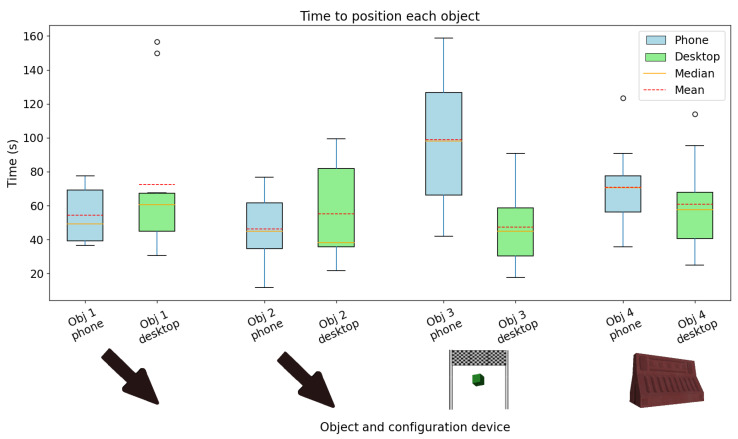
Average placement time for each virtual object used in the study. Blue boxplots represent the placements using the smartphone; green boxplots represent the placements using the computer.

We recruited 10 participants (2 females), with ages ranging from 19 to 34 (M = 24.8, SD = 4.61). From these, 30% had contact with AR prior to this study. All participants managed to conclude both tasks. The boxplots in Figure 7 summarize the efficiencies with both methods. At the beginning of the tasks, the participants were slightly faster at placing the first object using the smartphone. In the second object, participants were more efficient using the computer; however, some were still considerably slower. Regarding the last two objects, participants tended to be significantly quicker using the computer, indicating that using the computer, the effectiveness increased with less training time. Relative to the distance between the configurable virtual objects and the semi-transparent ones, the error was higher in every virtual object using the smartphone. Concerning the orientation error and the scaling error, the gathered results in each device were similar, obtaining similar errors for each object.

Regarding participant opinions, they considered it easier to place the virtual objects in the correct positions and scales than in the correct orientations. They agreed that, in both devices, their performances would improve with training, with 90% of them also reporting not needing the support of a technical person to create configurations. In general, participants preferred to use the smartphone over the computer. Applying the configuration using the computer, 30% were completely satisfied and 70% were satisfied; regarding the smartphone configuration tool, 60% were completely satisfied, 30% were satisfied, and 10% were unsatisfied.

Overall, participants were more efficient in configuring the environment using the computer. This device also showed higher accuracy when setting the virtual objects in the intended positions. This was justified by the familiarity of participants with using this device for such tasks. Using the implemented features, such as moving through the architectural plan using the mouse and zoom in/out, participants were faster than physically moving though the real environment, trying to achieve better accuracy, which even so, did not result in better accuracy, as this task was also harder to achieve in an immersive augmented reality environment. However, the smartphone was preferred by most participants, who reported that it was more interesting, entertaining, and stimulating.

### 3.3. User Study 2—Comparing Tracking Methods

**Figure 8:**
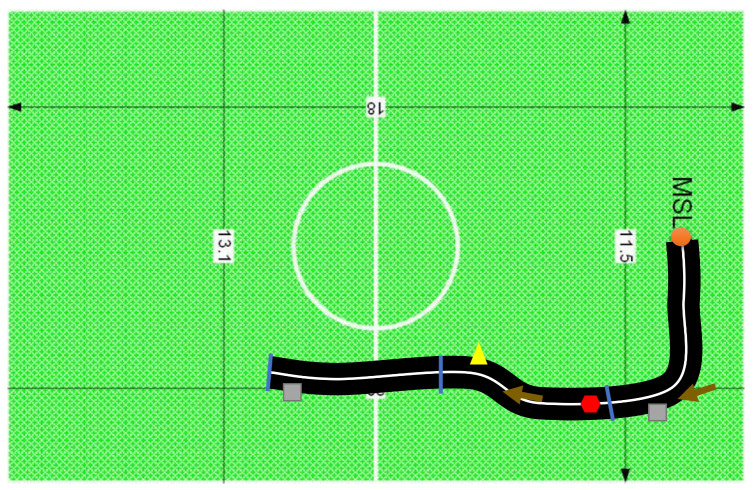
Schematic of the AR environment for the second user study. Description: The orange circle represents the starting position; the blue lines are the passage points; the brown arrows are directional arrow objects; the gray squares are obstacles; the yellow triangle is a spotlight; the red hexagon is a stop sign.

The goal of the second user study was to compare different tracking capabilities while the participants interacted with the game and controlled the wheelchair in the physical space, and to understand their preferences and possible difficulties. The smartphone used for this user study was the Samsung Galaxy S7. Two experimental conditions were considered: C1—uses operating mode O1 (motion tracking using cloud anchors) (youtube.com/watch?v=ue0Cl0Lxu0U&ab_channel=RafaMaio (accessed on 12 September 2022)); C2—uses operating mode O2 (offline motion tracking) (youtube.com/watch?v=fz6DtVK4Ibo&ab_channel=RafaMaio (accessed on 12 September 2022)). We configured the ARSG with three passage points, two obstacles, one stop sign, one spotlight, and two directional arrows (Figure 8). The tasks consisted of completing the ARSG track, passing by the three passage points in the shortest time possible, while complying with the remaining additional signs, and following the line and the moving car. As in the previous study, the orders of the tasks alternated among users. The following measures were considered: the time needed to complete one lap around the environment, logged in seconds by the device; the number of collisions with virtual obstacles; the number of successful stops; the number of exits from the main path. Moreover, participants’ preferences, level of entertainment, engagement, and satisfaction) were obtained through a post-task questionnaire. As for tracking, we categorized the technological behavior into one of four classifications:

- **Successful placement**: Every virtual object and the road appeared to be correctly placed, and any errors were minimal;
- **Placement with auto-correction**: The virtual objects and the road were misplaced, but the game-tracking corrected their positions, allowing to end the run;
- **Semi-successful placement**: The virtual objects and the road were not in the right place; however, it was clear which was the path, and no real obstacles were prevented from finishing the circuit;
- **Unsuccessful placement**: It was necessary to restart the game. It was impossible to finish the game because the virtual objects and the road were completely misaligned in relation to the configured AR environment.

Participants were instructed on the experimental setup, the tasks, and the robotic wheelchair, and gave their informed consent. Then, they were introduced to the ARSG. A time for adapting to the power wheelchair was provided until they were comfortable. In the end, participants answered a post-task questionnaire. A between-subjects experimental design was used, i.e., half of the participants performed the tasks using condition C1 and the other half C2.

#### Results and Discussion

We recruited 20 participants (5 female), with ages ranging from 19 to 34 (M = 20, SD = 3.58). Half of the participants participated in the configuration study. From these, 20% had contact with indoor navigation applications, 30% with AR, and none had experiences with regular or motorized wheelchairs prior to the study.

All participants concluded the tasks with success, passing by every passage point. On average, participants left the road once, caused 0.28 collisions, and took 33 s per run. In addition, 60% of the participants respected the stop sign. The distance between a specific point of the wheelchair and the line in the middle of the road during the entire track was around 0.33 meters and to the moving car around 0.40 m. Following the white line, participants adopted a faster speed level driving the wheelchair than chasing the moving car, leading to completing the track an average of 5 s faster.

Regarding users opinions, most participants considered the ARSG intuitive and easy to use. They agreed that their performances in the game would improve with training. With respect to the *’follow the line’* mode, 75% completely agreed that this facilitated interaction with the wheelchair, 20% agreed, and 5% had neutral opinions. Identical results were obtained in the *’follow the virtual car’* mode, where 75% completely agreed and 25% agreed. Comparing both conditions, they were equally appreciated, but pursuing the moving car was found to be more useful for learning to control the power wheelchair, with 35% preferring this version over the line (where 50% of the group had a neutral opinion). The preference for chasing the moving car with a constant speed was due to some participants learning that the control joystick had sensitivity, increasing/decreasing the wheelchair speed depending on the force applied to it, without changing the wheelchair speed level. As the moving car in the task had a slow speed, some users tried to adapt (learning this joystick sensitivity and how to benefit from it). The major difficulty while playing the ARSG consisted in following the road curves since it was harder to keep the wheelchair perfectly aligned with the middle road line, due to the smartphone image perspective. An interesting suggestion by the participants was that a small window located at the corner of the smartphone could display a map of the user’s location in space aiming to facilitate the understanding of mistakes while driving the wheelchair.

Overall, this study suggests that ARSG has potential in facilitating learning and interacting with a robotic wheelchair as it supports learning some of its features (e.g., joystick sensibility); participants could try to pass through tight spaces and avoid objects without being afraid of damaging the equipment and hurting themselves, while enjoying the time spent during the learning stage. Most users elected the *’follow the virtual car’* mode as more intuitive and useful for learning. However, the *’follow the line’* mode was considered to grant more freedom to participants, which makes it more entertaining; some of the participants had a deeper understanding of the spatial notion while using the wheelchair.

Finally, Table 1 summarizes the obtained results for the tracking methods. These results suggest that condition C1 has more robust and precise tracking, while condition C2 supports an easier way to configure the environment with fewer limitations. It was also observed that runs using condition C1 tend to be more successful as the ARCore technology, applying this mode, uses the visual features of the real environment to place the virtual content in relation to the stored points of interest; at any time, if a motion tracking error occurs, it can use real-world texture information (previously stored during the configuration) to reposition the misaligned virtual content and recalibrate its internal pose. As a final remark, participants who experienced unsuccessful placements considered it annoying to start the task from the beginning.

In summary, although both methods may be used for the intended purpose, participants selected motion tracking using the cloud anchors operating mode (O1) as the better option due to its capacity to start the ARSG anywhere and to maintain the virtual objects in place, correcting the produced drift with time. Despite this, the offline motion tracking operating mode (O2) can be a viable option as well, overcoming some limitations and negative aspects of operating mode O1. It allows easier and faster configurations, not requiring the placement of cloud anchors, which also enables the pervasive AR tool in locations without internet connections since there is no longer a need to access the API for storing and retrieving cloud anchors. Not using cloud anchors also permits larger changes in physical scenarios, which could lead to a poorer recognition of the anchored zone. In addition, it maintains a better relative position between virtual objects, as these do not suffer from cloud anchor adjustments. However, this operating mode (O2) has two main limitations: when starting the ARSG, the device must be in the same pose as the configuration when starting; this is the reference point for the virtual object placement. Moreover, the user’s continuous experience is more limited, since the motion tracking errors are not corrected, increasing the drift to a point that the application can no longer be used.

In fact, each operating mode can be advantageous depending on the use case. Considering a scenario in which an individual with motor disabilities would visit a museum, operating mode O1 would be ideal as virtual information would desirably rest near the corresponding real item and allow for revisiting exhibits. In contrast, operating mode O2 can be beneficial in scenarios suffering from daily/weekly modifications, e.g., a structure under construction, where an illustration of the intended result wants to be presented; however, the building expansion and the material disposition are rather volatile.

## 4. Conclusions

This work proposes an augmented reality serious game (ARSG) as a learning tool for the control of a robotic wheelchair by individuals with motor disabilities. Given that to experience the ARSG, a previous configuration must occur, a first study with 10 participants took place. The goal was to compare two methods: smartphone and computer regarding efficiency and accuracy. Participants preferred the smartphone, being considered more engaging, while the computer was characterized by better task performance.

A second study with 20 participants was conducted to compare the two operating methods for tracking purposes: O1—motion tracking using cloud anchors; O2—offline motion tracking. Results suggest that operating mode O1 is more robust, providing a better perception of the virtual content presented in the real world. In contrast, operating mode O2 has less accuracy, but a higher simplicity in the configuration process, as it does not need the placement of cloud anchors. Nevertheless, both operating modes appear to be valid alternatives (according to the scenarios and requirements addressed) for facilitating novel user learning/training processes.

This study is being expanded, i.e., by considering other serious games with different narratives, as well as multiple levels of complexity to understand if these further affect engagement. We also intend to explore a co-located collaborative setting, having more than one wheelchair user simultaneously moving in the physical space. Lastly, we expect to conduct a second user study with regular wheelchair users as participants (which was not possible due to the recent pandemic), which can lead to a more realistic level of validity and more pertinent feedback.

## Figures and Tables

**Table 1:** Comparison between the two conditions: C1—motion tracking using cloud anchors; C2—offline motion tracking. Results from 20 participants, half using operating mode C1 and the remaining half adopting operating mode C2.

Operating Mode	N° of Runs	Successful Placements (%)	Placements with ARCore Auto Corrections (%)	Semi-Successful Placements (%)	Unsuccessful Placements (%)
Condition C1	21	**61.9**	19.0	14.3	**4.8**
Condition C2	26	50.0	0.0	26.9	23.1

## Data Availability

The datasets generated during and/or analysed during the current study are available from the corresponding author upon reasonable request.
